# Supplementary material for: A major locus controls a biologically active pheromone component in Heliconius melpomene
Source: Evolution. 2020 Jan 20;74(2):349–64. doi: 10.1111/evo.13922 (PMC7027519; doi:10.1111/evo.13922)

**Supplemental Figures**

SI Figure 1: Comparison of the major compounds in *Heliconius melpomene* and *H. cydno*. Compounds shown are those contributing at least 1% of the total bouquet amount in either species. Numbers under each bar indicate how many samples (out of 31 for *H. melpomene* and 26 for *H. cydno*) the compound was found in. Significantly different compounds: * p < 0.05; ** p < 0.01; *** p < 0.001. n.s., not significant.


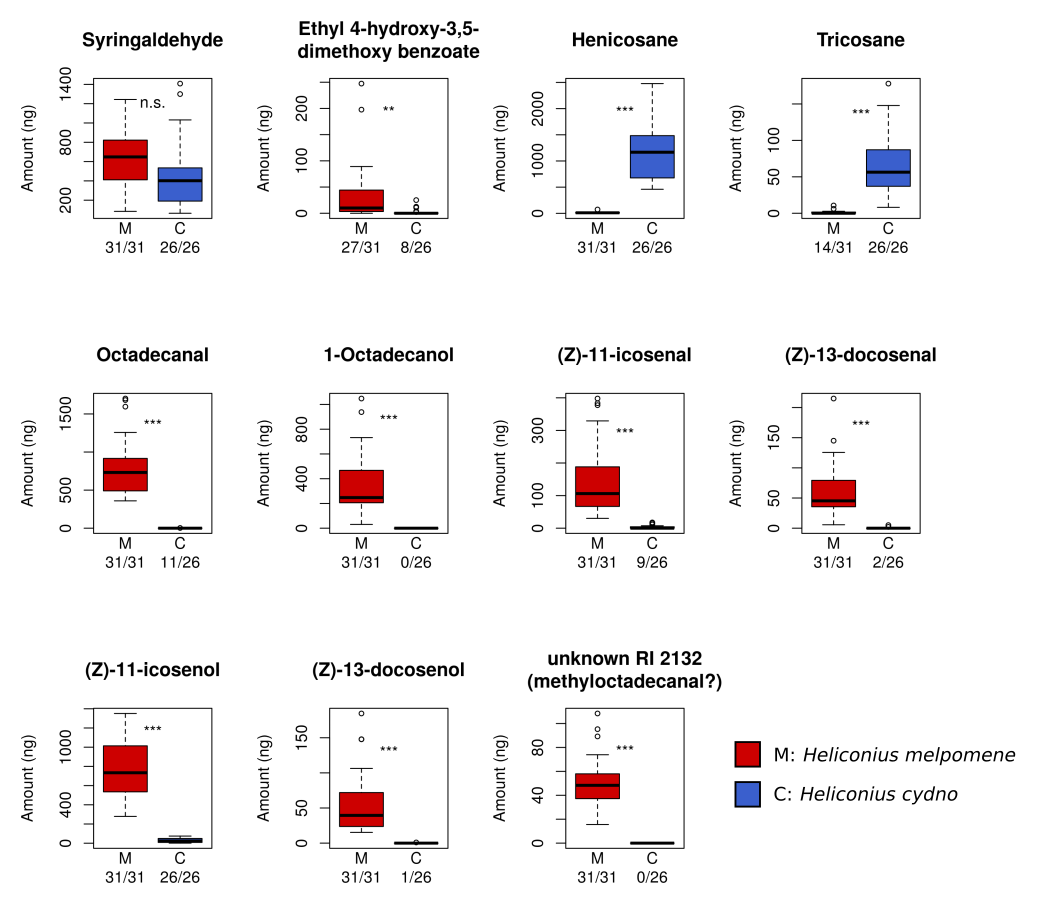


SI Figure 2: Absolute and relative abundance of different compound classes in *H. melpomene* and *H. cydno*. n.s., not significant; * p < 0.05; ** p < 0.01; *** p < 0.001. The two unknown categories were not tested as the compound types are not known.


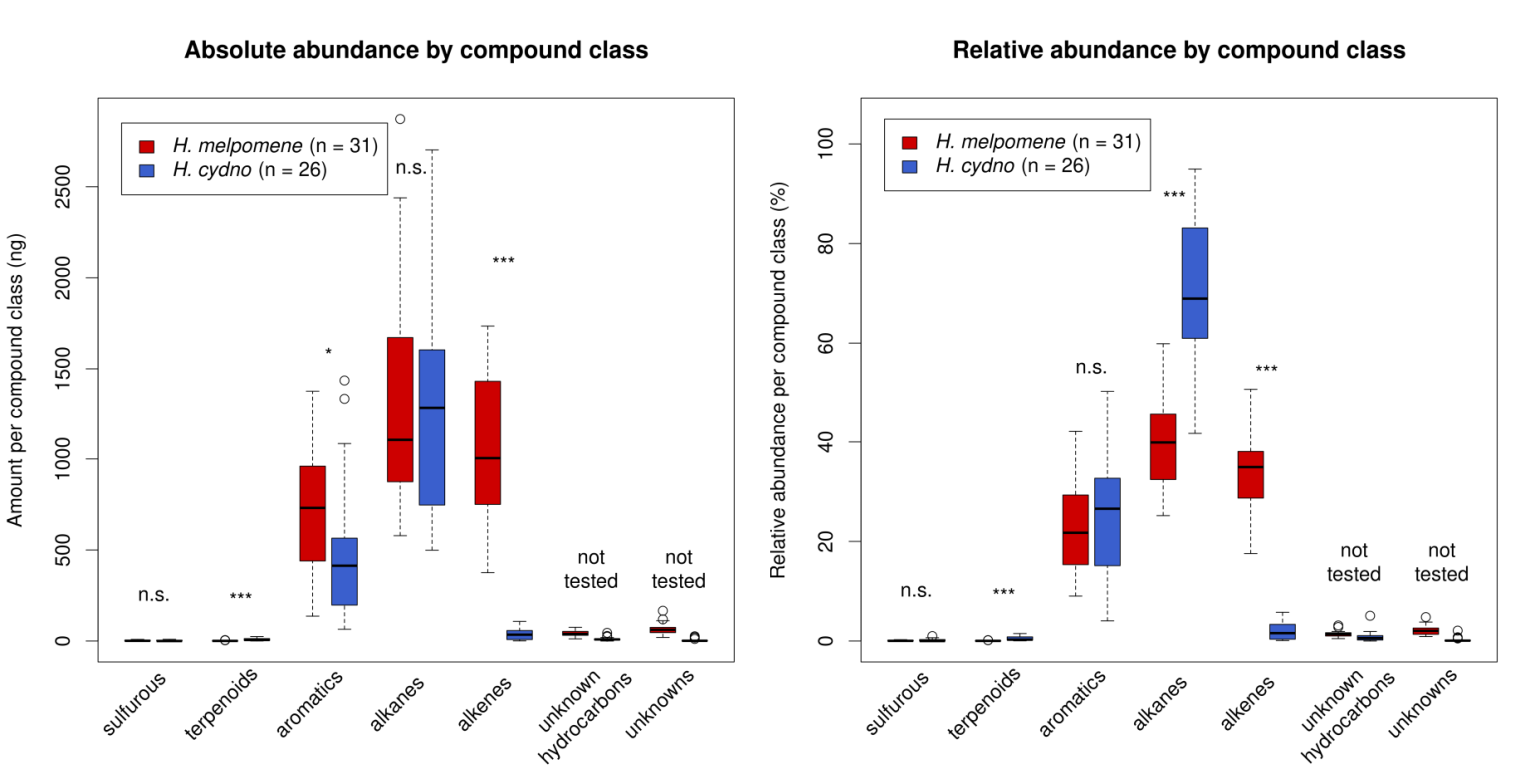


SI Figure 3: The seven compounds found in at least 0.1ng/mm^2^ of wing tissue in at least one wing region in *Heliconius cydno*. A: Compound abundance per square millimeter of tissue. B: Compound abundance without tissue area correction. Numbers under each bar indicate how many samples (out of eight) the compound was found in; letters above bars indicate significant differences between regions. n.s., not significant. A: hindwing androconia; O: forewing overlap region; H: hindwing excluding androconia; F: forewing excluding overlap region.


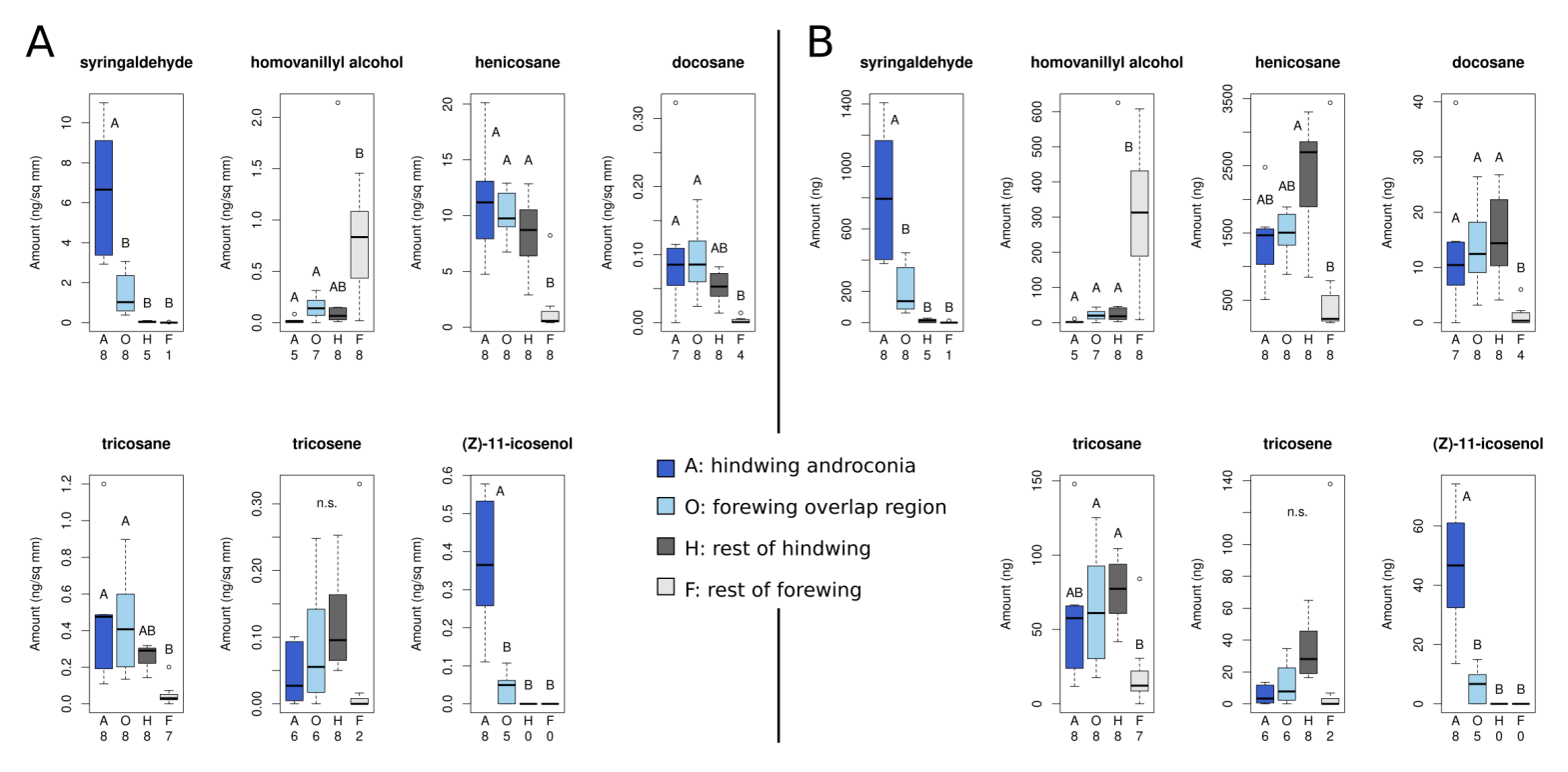


SI Figure 4: Structures and names of major components of the androconia of *H. melpomene* and *H. cydno* used in electrophysiological experiments.


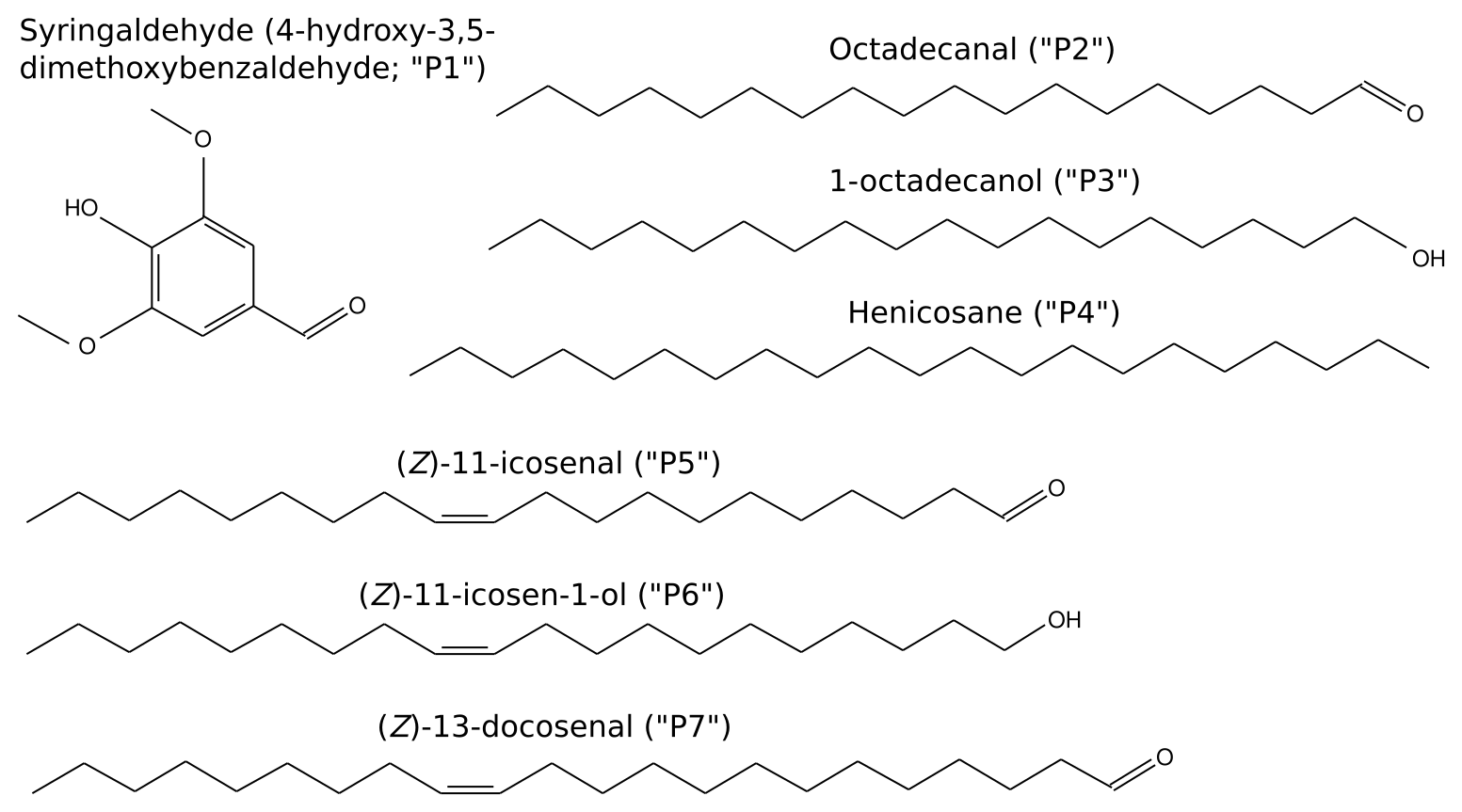


SI Figure 5: Synthesis of target compounds used in electrophysiological experiments. IBX: iodosobenzoic acid; LiAlH: lithium aluminum hydride.


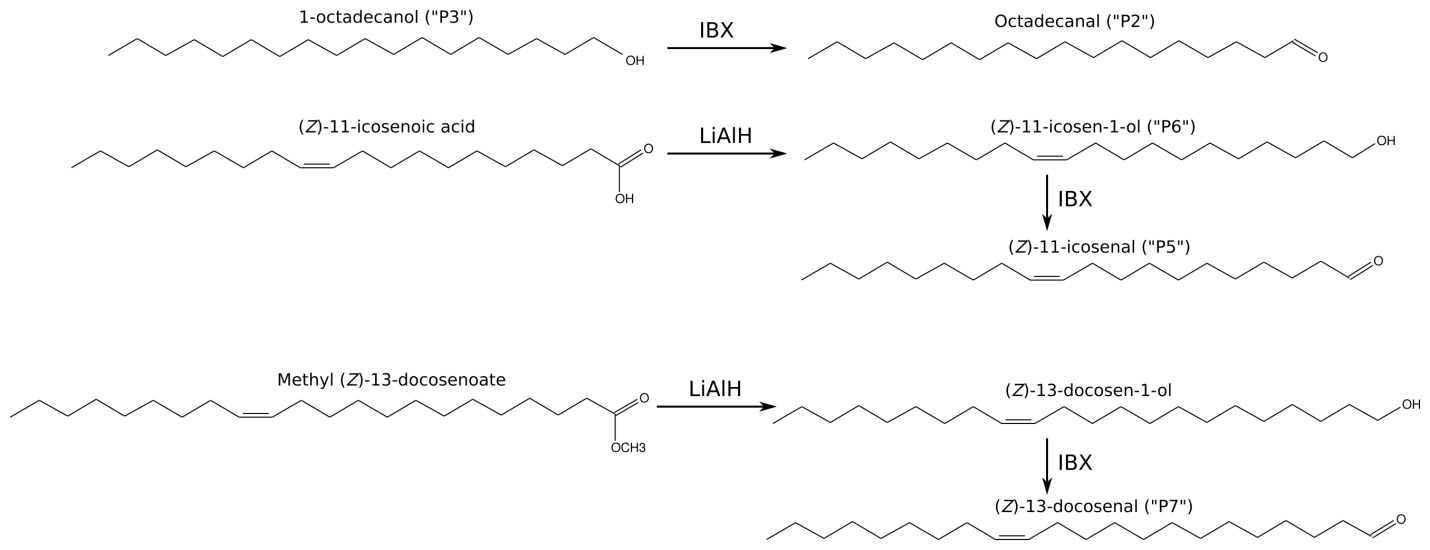


SI Figure 6: *Heliconius* *melpomene* responds to electrophysiological stimuli. Top to bottom: dichloromethane plus 2-tetradecyl acetate (internal standard) (negative control), *Lantana* extract (positive control), natural *H. melpomene* male wing extract, depiction of stimulus pulse timing. Data from a single virgin female. Bar-ended lines indicate the measured amplitude of the antennal response.


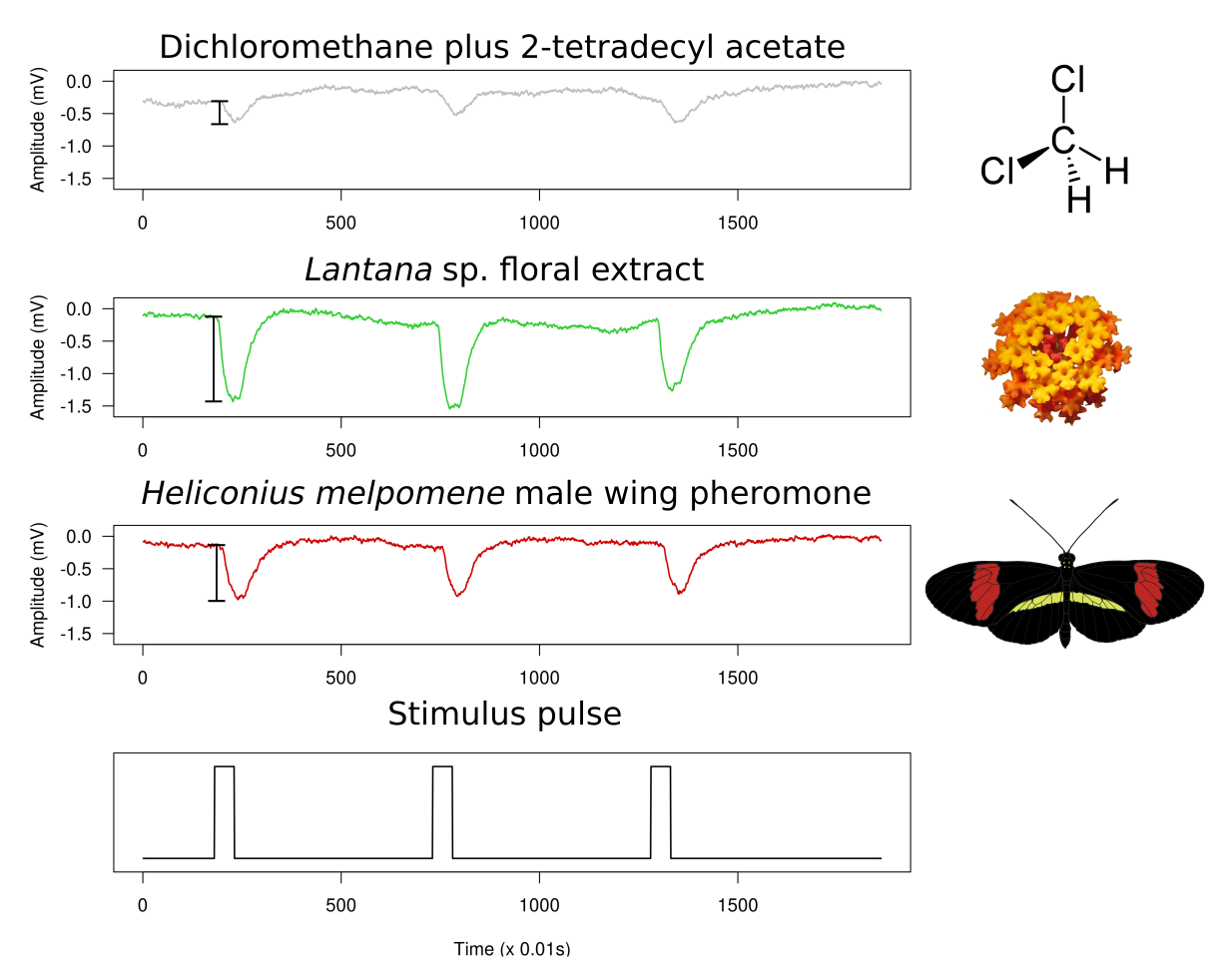


SI Figure 7: Long-term adaptation to natural and synthetic stimuli in *Heliconius* butterflies. The 95% confidence intervals of the robust LMM slope are shown; a negative slope means that responses to that stimulus drop over time. P1-P7: see Figure 2.


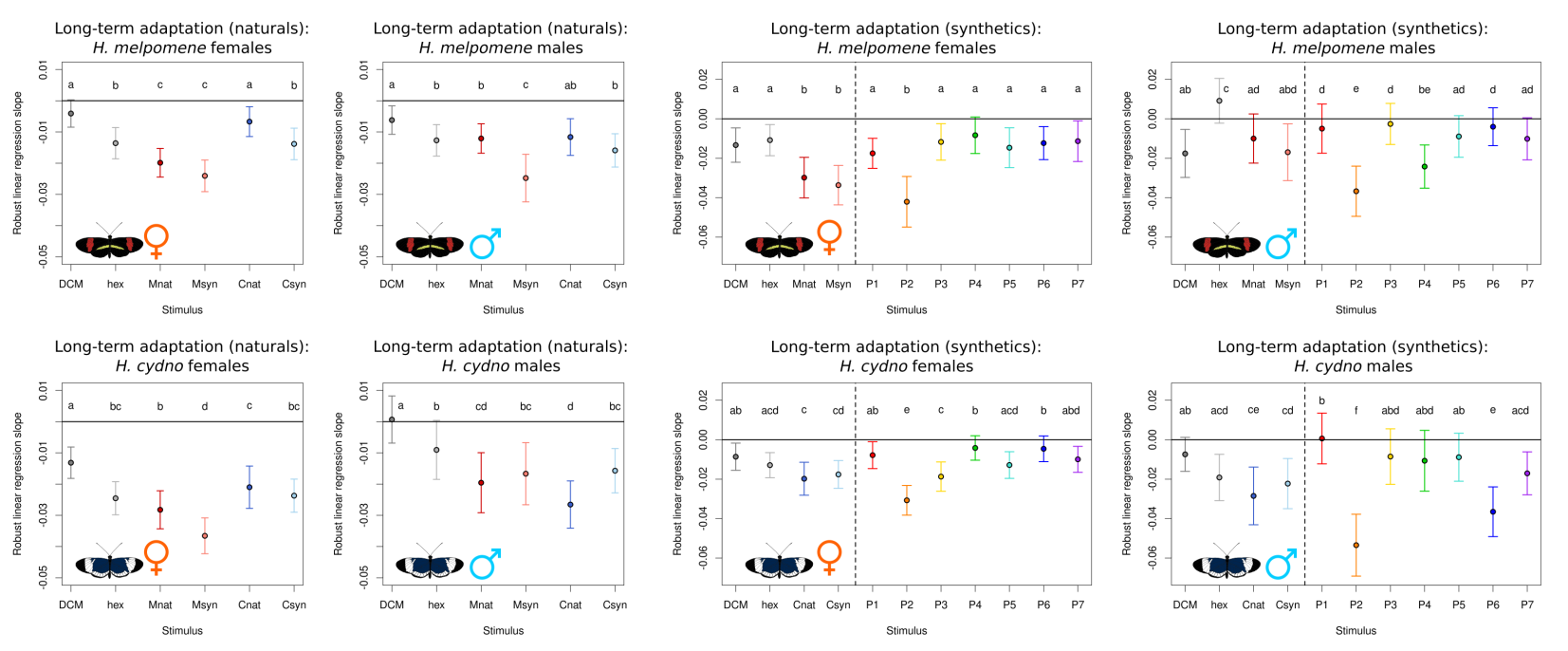


SI Figure 8: Strength of long-term adaptation correlates with amplitude of EAG response in a sex-specific fashion. In females, a stronger response to a given stimulus correlates with a stronger degree of LTA both overall and for the synthetic compound set. In males the same is seen overall and for the natural extract set in *H. cydno*, but not in *H. melpomene*.


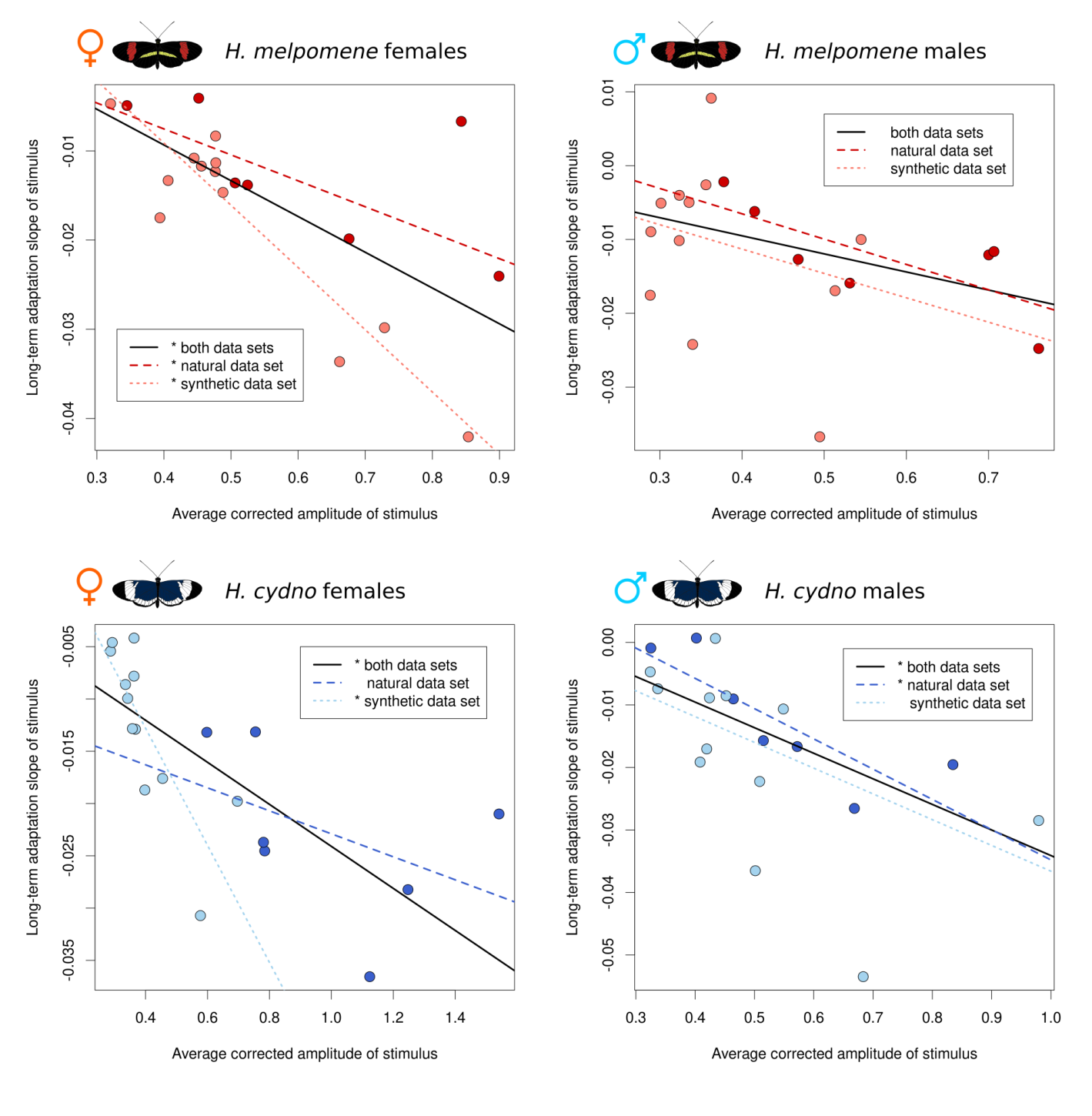


SI Figure 9: Octadecanal persistence in treated males over time. Bars show individual males, with two males per treatment-time point combination. m1-8: separate male individuals.


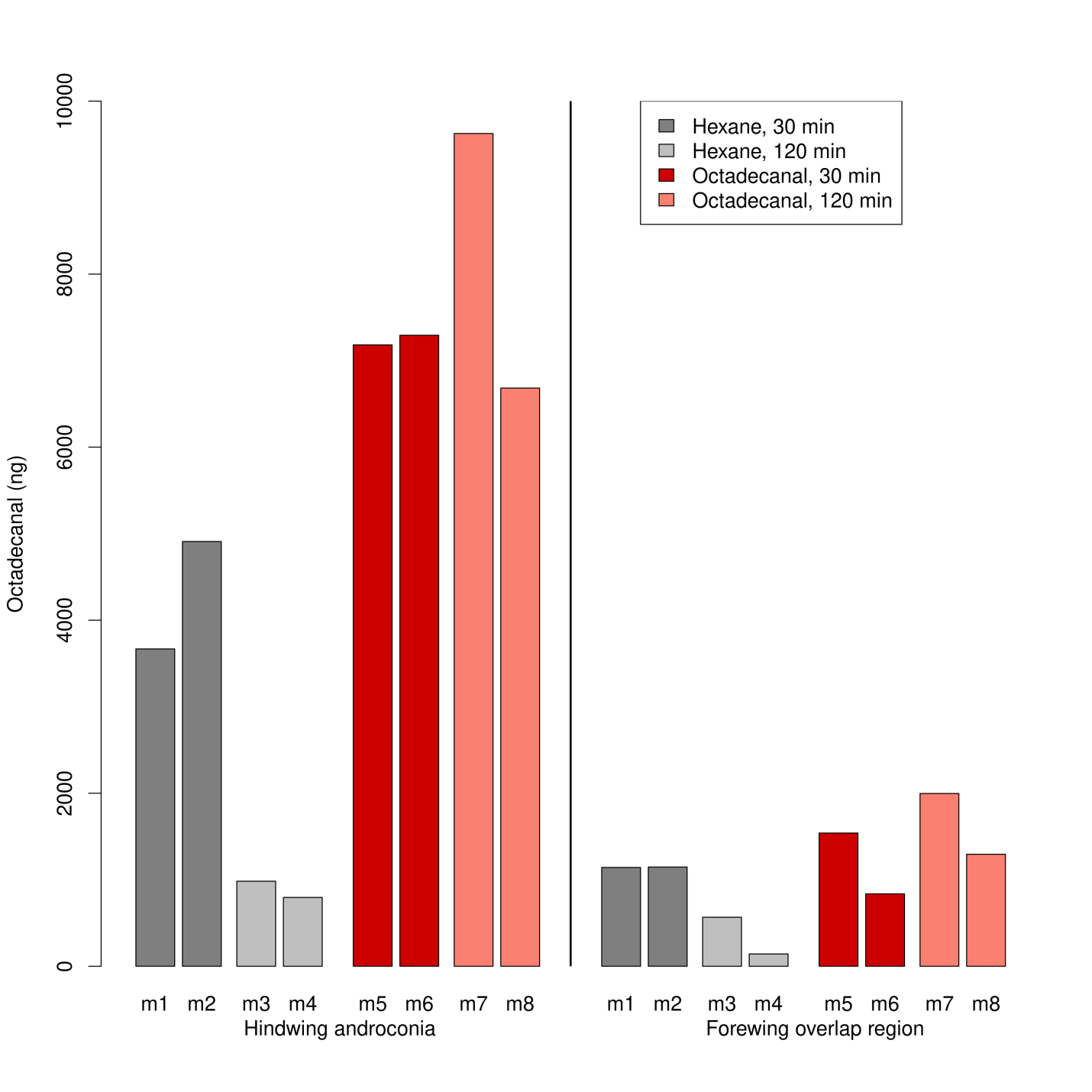


SI Figure 10: Octadecanal in *H. melpomene*, *H. cydno*, two F_1_ families (one in each crossing direction), and the ten backcross to *H. melpomene* families used in QTL mapping. Colors: blue (*H. cydno*); purple (F_1_ crosses of *H. melpomene* and *H. cydno*); pink (backcrosses to *H. melpomene*); red (*H. melpomene*).


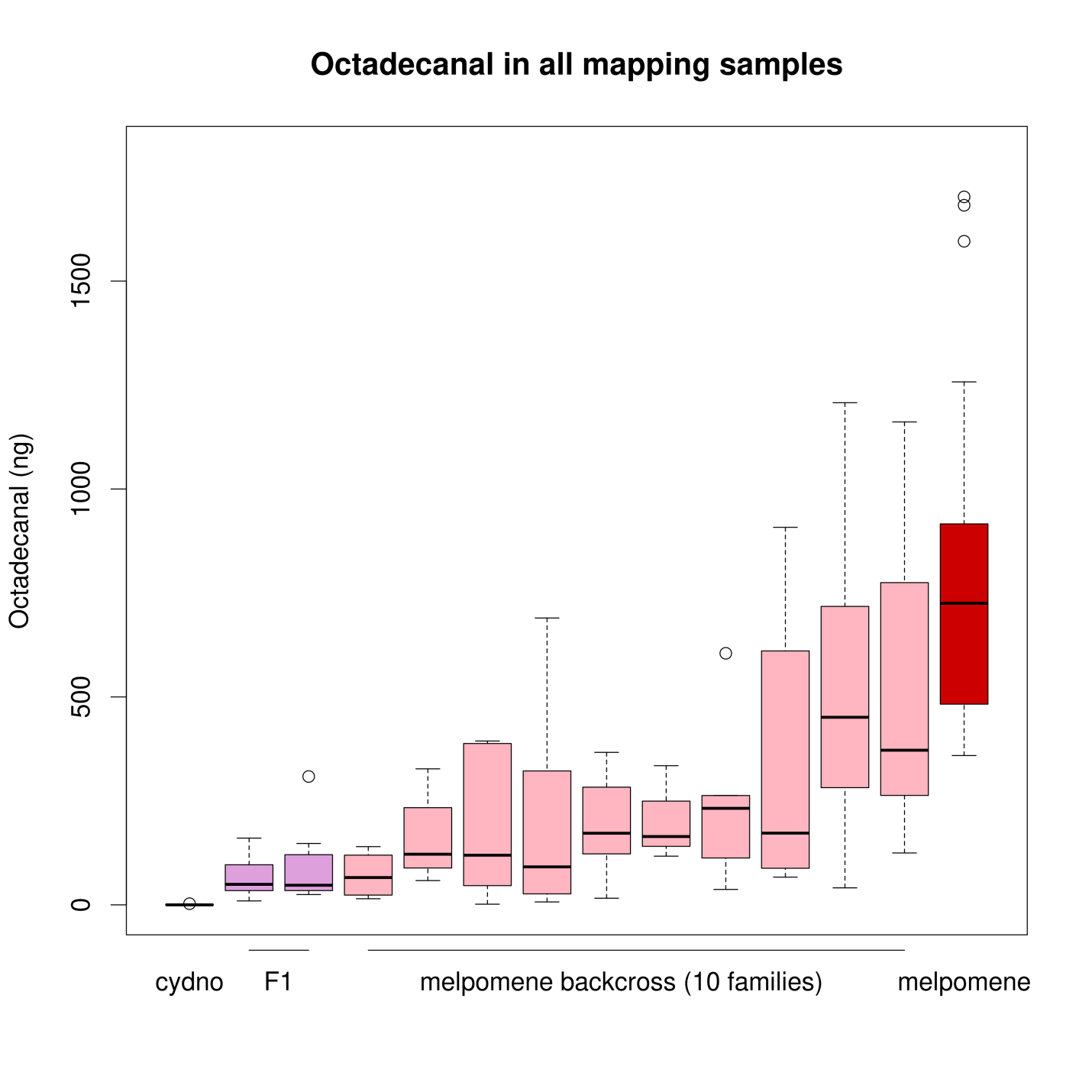


SI Figure 11: Chromosome 20 QTL map for production of octadecanal and octadecanol in *H. melpomene*. Shaded regions indicate the Bayesian confidence intervals with kinship structure taken into account and black line indicates the peak of the QTL.


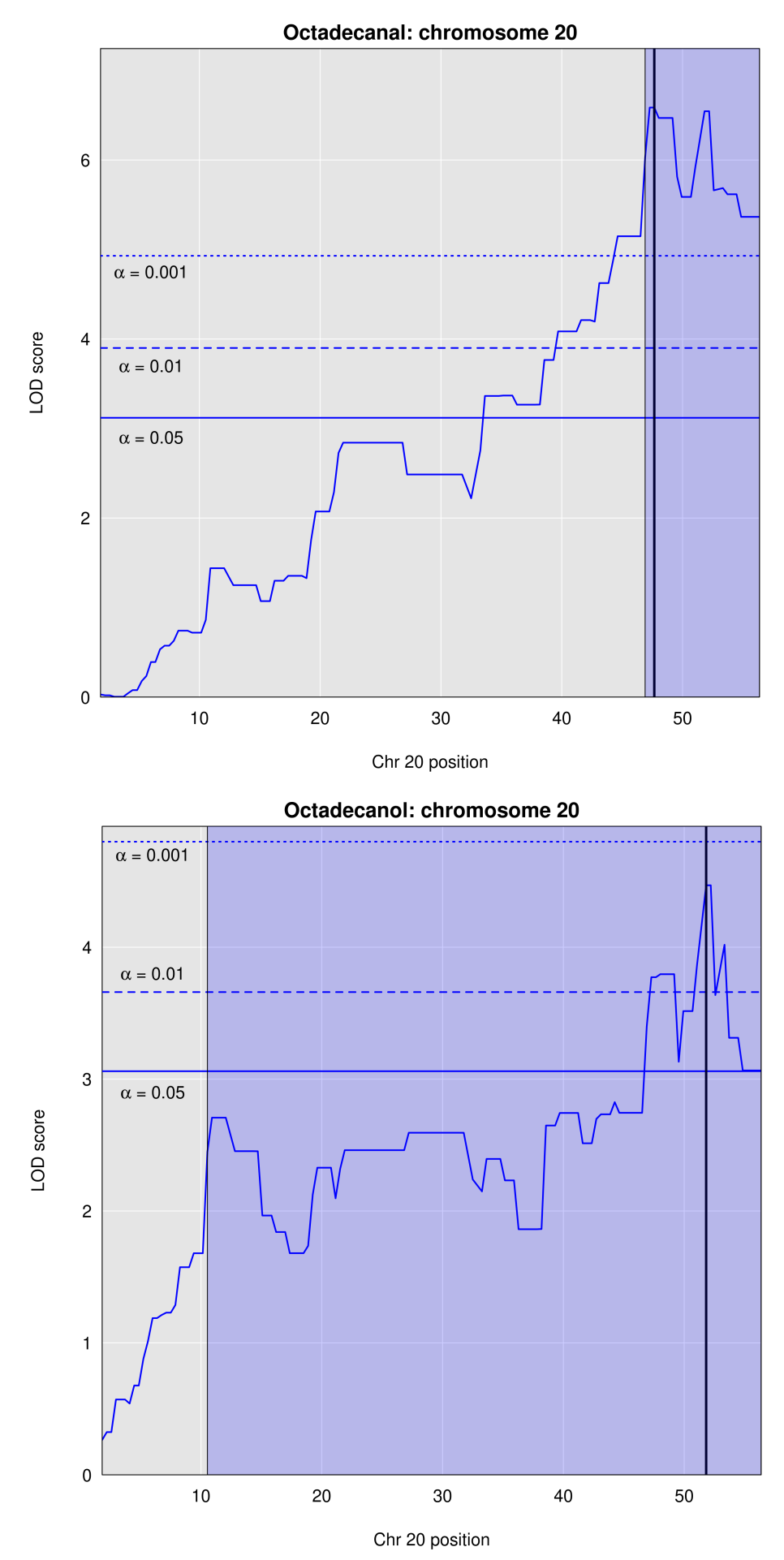

Supplement: Supplementary file 1 — SI Figure 1: Comparison of the major compounds in Heliconius melpomene and H. cydno. SI Figure 2: Absolute and relative abundance of different compound classes in H. melpomene and H. cydno. SI Figure 3: The seven compounds found in at least 0.1 ng/mm2 of wing tissue in at least one wing region in Heliconius cydno. SI Figure 4: Structures and names of major components of the androconia of H. melpomene and H. cydno used in electrophysiological experiments. SI Figure 5: Synthesis of target compounds used in electrophysiological experiments. IBX: iodosobenzoic acid; LiAlH: lithium aluminum hydride. SI Figure 6: Heliconius melpomene responds to electrophysiological stimuli. SI Figure 7: Long‐term adaptation to natural and synthetic stimuli in Heliconius butterflies. SI Figure 8: Strength of long‐term adaptation correlates with amplitude of EAG response in a sex‐specific fashion. SI Figure 9: Octadecanal persistence in treated males over time. SI Figure 10: Octadecanal in H. melpomene, H. cydno, two F1 families (one in each crossing direction), and the ten backcross to H. melpomene families used in QTL mapping. SI Figure 11: Chromosome 20 QTL map for production of octadecanal and octadecanol in H. melpomene. Shaded regions indicate the Bayesian confidence intervals with kinship structure taken into account and black line indicates the peak of the QTL. [file EVO-74-349-s001.docx]
